# 

**Published:** 1884-01

**Authors:** 


WHOLE NUMBER, CCLX. | JANUARY, 1884. | NUMBER 6, VOLUME XXIII.

LIBRARY INDEXED S. G. O.

### THE BUFFALO Medical and Surgical Journal.

EDITORS:

THOS. LOTHROP, M. D., A. R. DAVIDSON, M. D.,

P. W. VAN PEYMA, M. D.

PUBLISHED BY THE MEDICAL JOURNAL ASSOCIATION.

No. 5 WEST CHIPPEWA ST.

Entered at the Post-Office at Buffalo, N. Y., as Second-class Mail Matter.

